# Adaption of the *ex vivo* mycobacterial growth inhibition assay for use with murine lung cells

**DOI:** 10.1038/s41598-020-60223-y

**Published:** 2020-02-24

**Authors:** Hannah Painter, Satria A. Prabowo, Felipe Cia, Lisa Stockdale, Rachel Tanner, Samuel Willcocks, Rajko Reljic, Helen A. Fletcher, Andrea Zelmer

**Affiliations:** 1 Department of Infection Biology, London School of Hygiene and Tropical Medicine, Keppel Street, London, WC1E 7HT UK; 2 Oxford Vaccine Group, Department of Paediatrics, NIHR Oxford Biomedical Research Centre, University of Oxford, Oxford, OX4 2PG UK; 3 The Jenner Institute, University of Oxford, Old Road Campus Research Building, Roosevelt Drive, Oxford, OX3 7DQ UK; 4 Institute of Infection and Immunity, St George’s University of London, Cranmer Terrrace, London, SW17 0RE UK

**Keywords:** Tuberculosis, Vaccines

## Abstract

In the absence of a correlate(s) of protection against human tuberculosis and a validated animal model of the disease, tools to facilitate vaccine development must be identified. We present an optimised *ex vivo* mycobacterial growth inhibition assay (MGIA) to assess the ability of host cells within the lung to inhibit mycobacterial growth, including Bacille Calmette–Guérin (BCG) and *Mycobacterium tuberculosis* (MTB) Erdman. Growth of BCG was reduced by 0.39, 0.96 and 0.73 log_10_ CFU following subcutaneous (s.c.) BCG, intranasal (i.n.) BCG, or BCG s.c. + mucosal boost, respectively, versus naïve mice. Comparatively, a 0.49 (s.c.), 0.60 (i.n.) and 0.81 (s.c. + mucosal boost) log_10_ reduction in MTB CFU was found. A BCG growth inhibitor, 2-thiophenecarboxylic acid hydrazide (TCH), was used to prevent quantification of residual BCG from i.n. immunisation and allow accurate MTB quantification. Using TCH, a further 0.58 log_10_ reduction in MTB CFU was revealed in the i.n. group. In combination with existing methods, the *ex vivo* lung MGIA may represent an important tool for analysis of vaccine efficacy and the immune mechanisms associated with vaccination in the organ primarily affected by MTB disease.

## Introduction

Tuberculosis (TB) is the leading cause of death from a single infectious agent. In 2017, approximately 1.6 million individuals died from TB and 10 million fell ill with the disease^1^. The World Health Organization (WHO)’s End TB Strategy aims to reduce TB deaths by 95% and new cases by 90% between 2015 and 2035^2^. However, the current tools available for control of TB are inadequate and must be improved if this goal is to be met.

The Bacille Calmette–Guérin (BCG) vaccine remains the only clinically approved vaccine against TB. In high-burden countries the vaccine is administered at birth and protects against severe forms of TB in infants^3^. However, protection against pulmonary forms of the disease in adults varies dramatically between different geographical regions, with prior exposure to mycobacteria, either environmental mycobacteria or *Mycobacterium tuberculosis* (MTB) itself, thought to act as a major influence on this variance^4^.

Despite recent progression in the TB vaccine pipeline, with efficacy signals reported from two vaccines in phase 2b trial^5,6^, the development and validation of new TB vaccines remains slow and our understanding of the host immune response to MTB remains poor^7^. One of the reasons for this is that preclinical testing currently relies on head-to-head comparisons of vaccine candidates across a number of animal models. Progression through the preclinical pipeline is largely based on immunogenicity readouts which have been criticised for being oversimplified^8–10^, and challenge studies which are time consuming and costly, and require a large number of animals for sufficient statistical power. Challenge studies have historically relied on use of MTB laboratory strains to evaluate vaccine protection. However, differences in virulence, fitness and T-cell subset responses in animal models challenged with diverse clinical strains of MTB have been reported^11–13^, and there is therefore growing interest in preclinical testing of vaccines against MTB isolates representative of the global diversity of the MTB complex (MTBC).

As a potential solution to the growing need for a more rapid and cost-effective method for preclinical vaccine testing, interest in functional assays as readouts of vaccine efficacy has emerged. The mycobacterial growth inhibition assay (MGIA) has been used as an ‘*ex vivo* challenge model’ to analyse the summative capacity of a mixed population of *ex vivo* derived host cells to control mycobacterial growth after vaccination^14^. MTB challenge is not required as host cells are harvested from animals at the peak of the immune response following vaccination. The functional efficacy of the vaccine is then predicted by *ex vivo* co-culture of host cells with mycobacteria. Quantification of mycobacterial growth is predominantly performed using the BACTEC mycobacterial growth indicator tube (MGIT) system, but conventional colony forming unit (CFU) enumeration from culture on solid media has also been used^15^. The cost and study duration of the MGIA are lower than a comparative MTB challenge study, and animal welfare is also improved. Since each animal provides sufficient cells for multiple assay inputs, the MGIA offers the potential to analyse multiple lineages of the MTBC in parallel, using considerably fewer animals than a challenge study of the same design^16^. These concepts are in line with the ‘Refinement’ and ‘Reduction’ criteria defined by the UK National Centre for the 3Rs^17^.

To date, preclinical MGIA protocols have been established for use with splenocytes^18,19^ and bone-marrow-derived macrophages in mice^20^, as well as whole blood and peripheral blood mononuclear cells (PBMCs) for larger animal models^21,22^. The MGIA has been performed in tandem with MTB challenge in a number of murine studies to determine how *ex vivo* growth inhibition correlates with protection from *in vivo* infection with MTB. These methods have been reported to correlate at the group level^19,20,23^.

Protection against pulmonary TB must be demonstrated by candidate TB vaccines; therefore, to be an effective tool for TB vaccine testing, the MGIA should be able to evaluate protective immunity in the lung. To the best of our knowledge, an MGIA using lung cells from animal models of TB has not been reported. In this report, we present an optimised MGIA protocol for use with murine lung cells, to assess the ability of host lung cells to inhibit mycobacterial growth following vaccination. Following optimisation of host cell and bacterial input number, the lung MGIA was able to detect differences in both BCG (as a surrogate of MTB) and MTB Erdman growth inhibition between vaccine groups. Where residual BCG from immunisation was present in input cells, we found that use of the BCG inhibitor, 2-thiophenecarboxylic acid hydrazide (TCH), revealed additional MTB Erdman growth inhibition. In combination with current methods of preclinical TB vaccine assessment, the *ex vivo* lung MGIA could be used as a tool for analysis of vaccine efficacy and the underlying immune mechanisms associated with vaccination.

## Results

### Number of murine lung cells influences mycobacterial growth inhibition by immunised and control groups

A reduction in CFU burden after *in vivo* MTB challenge has been demonstrated in the murine lung in animals administered with BCG and/or various candidate TB vaccines compared with unvaccinated animals^23–25^. To determine whether the lung MGIA could be utilised as an ‘*ex vivo* challenge model’ to assess vaccine efficacy in animal models, C57BL/6 mice (six per group) received either BCG Pasteur Aeras subcutaneous (s.c.) or intranasal (i.n.) at week 0, BCG Pasteur Aeras s.c. at week 0 followed by an i.n. boost with the candidate vaccine spore-FP1 at week 3, or received no treatment. At six weeks, lungs were harvested, enzymatically digested and homogenised to generate a single cell suspension. Next, 3 × 10^6^ or 1 × 10^6^ lung cells from one pool of cells generated for each group were co-cultured with 100 CFU BCG Pasteur Aeras in 48-well plates. At 96 hours, cells were lysed and samples transferred to the BACTEC MGIT system until time to positivity (TTP) values were generated. BCG was used as a surrogate for MTB in early assay development as MGIA outcomes have previously been shown to correlate^21,26^.

**Figure 1:**
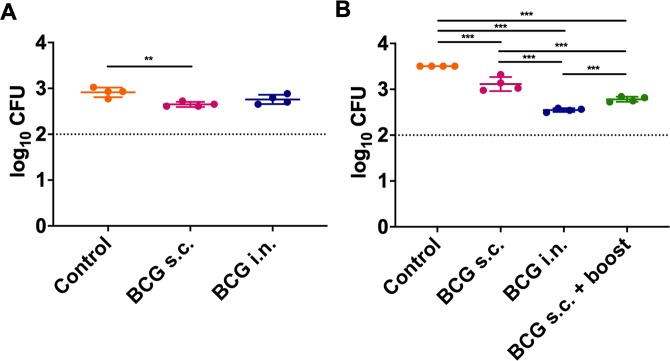
Host-cell density affects growth inhibition in the *ex vivo* MGIA. C57BL/6 mice (n = 6/group) received s.c. or i.n. BCG Pasteur Aeras at week 0 or no treatment (control). An additional group shown in part B received s.c. BCG Pasteur Aeras at week 0 followed by i.n. boosting with the candidate vaccine spore-FP1 at week 3. At six weeks, (**A**) 3 × 10^6^ or (**B**) 1 × 10^6^ lung cells were co-cultured with 100 CFU BCG Pasteur Aeras in 48-well plates. At 96 hours, samples were lysed and transferred to the BACTEC system until TTP values were generated. TTP values were converted to log_10_ CFU based on a standard curve. Data points (n = 4/group) represent samples generated from pooled cells isolated from six mice. Dotted line indicates mycobacterial input at day 0. Statistical significance was tested by one-way ANOVA followed by Tukey’s multiple comparison test. Error bars represent mean ± standard deviation. **p < 0.01 (adjusted); ***p < 0.001 (adjusted). ANOVA, analysis of variance; BCG, Bacille Calmette-Guérin; CFU, colony-forming units; i.n., intranasal; MGIA, mycobacterial growth inhibition assay; s.c., subcutaneous; TTP, time to positivity.

Initially, the lung MGIA was performed with 3 × 10^6^ lung cells (Fig. 1A), revealing a significant but small reduction (Δ0.26 log_10_ CFU) in growth following BCG s.c. vaccination compared with control animals (p = 0.0075; one-way ANOVA followed by Tukey’s multiple comparison test). No differences in BCG growth were found between the BCG i.n. group and control.

Decreasing the input lung cell count to 1 × 10^6^ resulted in improved sensitivity to detect significant reductions in growth of BCG in all vaccinated groups compared with the control group (Δ log_10_ CFU: BCG s.c. 0.39, BCG i.n. 0.96, BCG s.c. + mucosal boost 0.73; p ≤ 0.0001; one-way ANOVA followed by Tukey’s multiple comparison test) (Fig. 1B). When comparing BCG i.n. to s.c., a 0.57 log_10_ reduction in CFU was noted when vaccinating intranasally (p < 0.0001). Mucosal boosting with spore-FP1 three weeks following BCG s.c. was also found to reduce BCG growth by a further 0.33 log_10_ CFU compared with BCG s.c. alone (p = 0.0006).

Since *ex vivo* mycobacterial growth inhibition following BCG immunisation has previously been demonstrated in murine splenocytes^18,19,27^, an MGIA using splenocytes from the same animals was performed as a positive control in parallel with the lung MGIA. Using 3 × 10^6^ splenocytes co-cultured with 100 CFU BCG Pasteur, a 1.04, 0.86 and 0.87 log_10_ CFU reduction in BCG growth was observed in the BCG s.c., i.n. and BCG s.c. + mucosal boost groups respectively, compared with the control (p < 0.0001; one-way ANOVA followed by Tukey’s multiple comparison test) (Supplementary Fig. S1).

### Comparison of MTB Erdman and BCG growth inhibition

**Figure 2:**
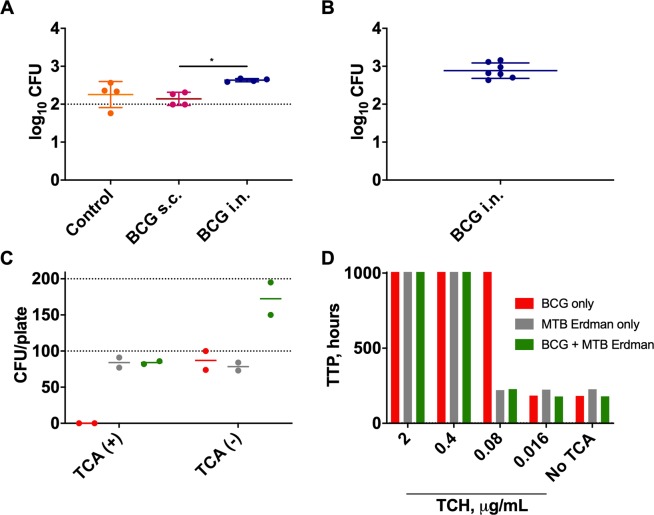
Residual BCG in murine lung cells following intranasal vaccination in the *ex vivo* MGIA. (A) C57BL/6 mice (n = 6/group) received s.c. or i.n. BCG Pasteur Aeras at week 0 or no treatment (control). At six weeks, 1 × 10^6^ lung cells were co-cultured with 100 CFU MTB Erdman in 48-well plates. At 96 hours, samples were transferred to the BACTEC system until TTP values were generated. TTP values were converted to log_10_ CFU based on a standard curve. Data points (n = 4/group) represent samples generated from pooled cells from six mice. Statistical significance was tested by one-way ANOVA followed by Tukey’s multiple comparison test. *p < 0.05 (adjusted). (B) Residual BCG in 1 × 10^6^ murine lung cells six weeks following immunisation with i.n BCG Pasteur Aeras was quantified using the BACTEC MGIT system. Data points represent four pools of cells from four individual mouse experiments (run as duplicate or single). (C and D) 100 CFU BCG Pasteur Aeras or MTB Erdman, or 100 CFU BCG Pasteur Aeras and 100 CFU MTB Erdman were: (C) plated on 7H11 agar plates in the presence or absence of 2 μg/mL TCH to confirm the inhibitory properties of TCH on BCG only (n = 2/condition); (D) added to MGITs in the absence or presence TCH (five-fold serial dilutions of 2 μg/mL stock: 0.4, 0.08, 0.016 μg/mL) to determine the optimal concentration of TCH to inhibit BCG growth only (n = 1/condition) in the BACTEC system. A TTP value of 1,008 hours (42 days) indicates a negative result (no bacteria detected). Dotted lines indicate mycobacterial input. Error bars represent mean ± standard deviation. ANOVA, analysis of variance; BCG, Bacille Calmette-Guérin; CFU, colony-forming units; i.n., intranasal; MGIA, mycobacterial growth inhibition assay; MGIT, mycobacterial growth indicator tube; s.c., subcutaneous; TCH, 2-thiophenecarboxylic acid hydrazide; TTP, time to positivity.

Next, to determine whether BCG growth inhibition identified in the vaccinated groups above correlates with growth inhibition of MTB Erdman, an MGIA was performed using the same host cell and bacterial input (Fig. 2A). Surprisingly, no differences in MTB growth were found between the vaccinated and control groups. In contrast, in the BCG i.n. group, a significant increase in MTB Erdman CFU of 0.49 log_10_ was noted compared with the BCG s.c. group (p = 0.03; one-way ANOVA followed by Tukey’s multiple comparison test). When comparing the control and BCG i.n. groups, a trend in increased growth in the i.n. group was observed; however this was not significant. Intra-assay variability was determined by calculating the coefficient of variation (CV): [standard deviation/mean] × 100. The CV quantifies the spread of data within each experimental group. When using 100 CFU of MTB Erdman in the MGIA, the CV for control and BCG s.c. was found to be 7.17% and 3.52%, respectively (Supplementary Table S1). Of note, the CV in the BCG i.n. group was 0.95%.

The lack of growth inhibition of MTB led us to hypothesis that residual live BCG from vaccination persisted in *ex vivo* isolated lung cells, potentially confounding CFU enumeration of MTB. To clarify this, 1  × 10^6^ lung cells only were added to supplemented MGITs in the absence of co-culture with mycobacteria to quantify residual BCG at six weeks following vaccination using the BACTEC MGIT system. In the BCG i.n. group, residual BCG at six weeks post-vaccination was 2.87 log_10_ CFU (mean calculated from four individual mouse experiments [run as duplicate or single]; maximum, 3.161; minimum, 2.634; Fig. 2B). No residual BCG was detected in samples from the BCG s.c. group (data not shown). To eliminate the confounding effect of residual BCG on MTB growth inhibition, we refined the assay further by addition of TCH, a compound known to specifically inhibit BCG growth.

### *Mycobacterium* spp. TCH susceptibility

At defined concentrations, TCH inhibits growth of BCG but not MTB^28^. Addition of 2 μg/mL TCH to 7H11 agar plates is standard practice in MTB challenge studies which include i.n. BCG vaccination^29,30^. The inhibitory properties of TCH on BCG, but not MTB Erdman, were confirmed here by plating 100 CFU of each of BCG Pasteur Aeras or MTB Erdman alone, or 100 CFU of BCG Pasteur Aeras and 100 CFU of MTB Erdman together on 7H11 agar plates in the presence or absence of 2 μg/mL TCH (Fig. 2C). MTB Erdman CFU was unchanged in the absence and presence of 2 μg/mL TCH. By contrast, BCG did not grow in the presence of 2 μg/mL TCH, indicating that enumeration of MTB Erdman only could be performed using this concentration of TCH in agar plates.

To date, use of TCH in the BACTEC MGUT system has not been reported. Therefore, 100 CFU of each of BCG Pasteur Aeras or MTB Erdman alone, or 100 CFU of BCG Pasteur Aeras and 100 CFU MTB Erdman together were added to supplemented MGITs in the presence (five-fold serial dilutions of a 2 μg/mL stock: 0.4, 0.08, 0.016 μg/mL) or absence of TCH to determine the optimal concentration of TCH to inhibit growth of BCG, but not MTB Erdman, in the BACTEC system (Fig. 2D). A concentration of 0.08 μg/mL TCH was found to inhibit growth of BCG only. Growth inhibition of both MTB Erdman and BCG was observed at TCH concentrations of 2 and 0.4 μg/mL, and no growth inhibition of either mycobacterial species was found at 0.016 μg/mL.

### Vaccine-mediated inhibition of mycobacterial growth in lung cell culture

Incorporating the optimised parameters described, an MGIA was performed to determine the vaccine-induced capacity of murine lung cells to control the growth of MTB Erdman. In addition to the aforementioned parameters, the mycobacterial input for the MTB Erdman MGIA was increased to 200 CFU. This was because residual BCG was not identified in lungs harvested from animals receiving BCG s.c., and yet no significant difference in growth inhibition of MTB Erdman was noted in the BCG s.c. group compared with control. Notably, intra-assay variability (CV) in the control and BCG s.c. groups was high using 100 CFU MTB Erdman (Supplementary Table S1). We therefore hypothesised that increasing the mycobacterial input may also improve the performance of the assay and decrease variability.

**Figure 3:**
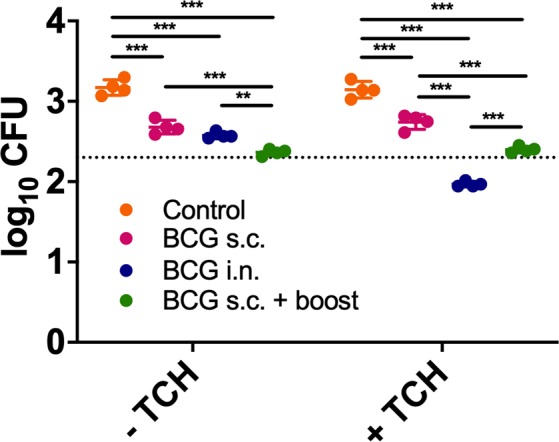
Optimised *ex vivo* MGIA comparing MTB Erdman growth inhibition conferred by vaccination in murine lung. C57BL/6 mice (n = 6/group) received s.c. or i.n. BCG Pasteur Aeras at week 0, s.c. BCG Pasteur Aeras at week 0 and i.n. boosting with the candidate vaccine spore-FP1 at week 3, or received no treatment (control). At six weeks, 1 × 10^6^ lung cells were co-cultured with 200 CFU MTB Erdman in 48-well plates. At 96 hours, samples were transferred to the BACTEC MGIT system until TTP values were generated. Mycobacterial quantification was performed in the presence and absence of TCH. TTP values were converted to log_10_ CFU based on a standard curve. Data points (n = 4/group) represent samples generated from pooled cells isolated from six mice. Dotted line indicates mycobacterial input at day 0. Statistical significance was tested by one-way ANOVA followed by Tukey’s multiple comparison test. Error bars represent mean ± standard deviation. *p < 0.05 (adjusted); **p < 0.01 (adjusted); ***p < 0.001 (adjusted). ANOVA, analysis of variance; BCG, Bacille Calmette-Guérin; CFU, colony-forming units; i.n., intranasal; MGIA, mycobacterial growth inhibition assay; MGIT, mycobacterial growth indicator tube; s.c., subcutaneous; TCH, 2-thiophenecarboxylic acid hydrazide; TTP, time to positivity.

Using the same four-group study design described above for the BCG MGIA, lungs were harvested at six weeks after vaccination and 1 × 10^6^ lung cells from pools of cells generated for each group were co-cultured with 200 CFU MTB Erdman in 48-well plates. At 96 hours, samples were lysed and transferred to the BACTEC MGIT system (Fig. 3) or plated on solid media (Supplementary Fig. S2), in the absence and presence of TCH.

Using the BACTEC MGIT system in the absence of TCH, significant reductions in growth of MTB Erdman were observed in all vaccinated groups compared with the control group (Δ log_10_ CFU: BCG s.c. 0.49, BCG i.n. 0.60, BCG s.c. + mucosal boost 0.81; p < 0.0001; one-way ANOVA followed by Tukey’s multiple comparison test). Mucosal boosting three weeks following BCG s.c. was found to reduce MTB Erdman growth by a further 0.32 log_10_ CFU compared with BCG s.c. alone (p = 0.0002). No significant differences in growth inhibition between the BCG s.c. and i.n. groups were identified. In the presence of TCH, reductions in growth of MTB Erdman were again found in all vaccinated groups compared with controls (Δ log_10_ CFU: BCG s.c. 0.40, BCG i.n. 1.18, BCG s.c. + mucosal boost 0.75; p < 0.0001), as well as a further reduction in growth associated with BCG s.c. + mucosal boost compared with BCG s.c. alone (Δ0.35 log_10_ CFU; p = 0.0001). Of note, a further reduction in MTB Erdman growth of 0.58 log_10_ CFU was noted in the BCG i.n. group compared with samples without TCH. Intra-assay variability was low in all groups, with a 4.53% and 1.38% reduction in CV in the control and BCG s.c. groups respectively when increasing MTB Erdman to 200 CFU (Supplementary Table S1).

When plating lysates on solid media (Supplementary Fig. S2) in the absence of TCH, significant reductions in growth of MTB Erdman were also noted in all vaccinated groups compared with the control group (Δ log_10_ CFU: BCG s.c. 0.34 [p = 0.0362], BCG i.n. 0.37 [p = 0.0210], BCG s.c. + mucosal boost 1.05 [p < 0.0001]; one-way ANOVA followed by Tukey’s multiple comparison test). In the presence of TCH, reductions in growth of MTB Erdman were found in the BCG s.c. (Δ0.37 log_10_ CFU; p = 0.0331), BCG i.n. (Δ 1.02 log_10_ CFU; p < 0.0001) and BCG s.c. + mucosal boost compared with the control group (Δ0.61 log_10_ CFU; p = 0.001). Again, a further reduction in MTB Erdman growth of 0.65 log_10_ CFU was noted in the BCG i.n. group compared with in the absence of TCH.

An MGIA using splenocytes from the same animals was performed as a positive control in parallel with the lung MGIA. Using 3 × 10^6^ splenocytes co-cultured with 200 CFU MTB Erdman, a 1.12, 0.93 and 1.30 log_10_ CFU reduction in MTB Erdman growth was observed using the BACTEC MGIT system in the BCG s.c., i.n. and BCG s.c. + mucosal boost groups respectively, compared with the control (p < 0.0001; one-way ANOVA followed by Tukey’s multiple comparison test) (Supplementary Fig. S3).

Using the optimised MGIA conditions described, variations in the ability of *ex vivo* derived host lung cells to inhibit MTB Erdman growth were found following vaccination. These variations were identified using the BACTEC MGIT system and plating on solid media. Use of the BCG inhibitor, TCH, revealed additional MTB Erdman growth inhibition in groups in which residual BCG was present six weeks following vaccination.

## Discussion

The MGIA has been developed as a tool to facilitate preclinical evaluation of TB vaccines using mycobacterial growth inhibition as a surrogate readout of vaccine-induced protection against TB^14^. To date, murine MGIAs have been performed using splenocytes and bone-marrow-derived macrophages, with mycobacterial growth inhibition *ex vivo* reported to correlate with *in vivo* protection in MTB challenge studies at the group level^18–20^.

TB is predominantly a pulmonary infection, and MTB challenge studies commonly enumerate bacterial burden in the lung and spleen of infected animals. We therefore aimed to develop a lung MGIA as a complementary tool for the preclinical evaluation of existing and experimental TB vaccines. Induction of an earlier, pulmonary, vaccine-induced immune response to infection is a key objective in TB vaccine development, highlighting the importance of development of tools able to analyse this compartment. To date, mycobacterial growth control of cells from human alveolar lavage^31^ and alveolar macrophages^32^ has been investigated using the MGIA. To the best of our knowledge, an MGIA using total lung cells from animal models of TB has not been reported.

In this study, an MGIA using BCG as a surrogate for MTB was performed in 48-well plates to determine whether this culture method is conducive to assessment of the control of mycobacterial growth by lung cells. Recent MGIA publications have recognised that host cell viability following co-culture in rotating screw-cap tubes is low, hypothesising that the lack of oxygen availability in the tubes and shear forces associated with rotation may be deleterious to cell viability^18,33^. Use of static 48-well plates in the splenocyte MGIA has been reported previously^34^. A mouse study was designed which included s.c. and i.n. administration of BCG, as well as a control unvaccinated arm. The experimental prime-boost vaccine, spore-FP1, was also included at later stages of assay development.

The majority of published MGIA studies use biological replicates (albeit using inbred mice) within each group. In 2016, Yang *et al*. presented a simplified splenocyte MGIA using five technical replicates per group from three pooled mice. Pooling was recognised as a limitation; however, it was found that by reducing variability, sensitivity to detect differences in growth inhibition between groups was improved. MGIA outcomes were reported to correlate with *in vivo* protection^34^. The simplified MGIA was performed in the current study using four technical replicates from each group of six pooled mice.

To date, 5 × 10^6^ splenocytes have been used in murine MGIAs^18–20^. When comparing host cell input numbers in the MGIA using rotating tubes, a higher number of splenocytes (5 × 10^6^) was found to increase sensitivity to differences in growth inhibition and improve intra-assay variability compared with lower inputs^19^. However, when using 48-well plates in the current study, higher host cell density resulted in considerable yellowing of the media at 96 hours (microscopic evaluation) indicative of nutrient exhaustion and potentially less healthy cells. To this end, we selected a lower input cell number for MGIAs performed in this study, with the aim to improve host cell viability and subsequent inhibitory capacity.

As a positive control during assay development, a splenocyte MGIA was run in tandem with the lung MGIA. Significant growth inhibition of BCG was observed in all vaccinated groups compared with the control using 3 × 10^6^ splenocytes and 100 CFU BCG (Supplementary Fig. S1). In comparison, when using lung cells, a modest reduction in mycobacterial growth was observed only in the s.c. group (vs control group) under the same conditions (Fig. 1A). No differences were observed between other groups. By contrast, reducing the lung cell input to 1 × 10^6^ resulted in a significant reduction in BCG growth in all vaccination groups (Fig. 1B), consistent with *in vivo* protection reported in the literature^24,30^.

To enable better comparison to *in vivo* MTB challenge experiments, an MGIA was performed using MTB Erdman with the same host and mycobacterial input as the BCG MGIA. Paradoxically, mycobacterial growth was increased in the BCG i.n. group compared with the BCG s.c. and the control group. No other growth differences were noted (Fig. 2A). We therefore sought to further optimise the MGIA conditions.

As advised by Zelmer *et al*.^19^, ‘direct-to-MGITs’ (host cells added directly to MGITs) for all experimental groups receiving a live vaccine were performed for all MGIAs. We report that six weeks following i.n. BCG immunisation, 2.87 log_10_ CFU BCG were present in 1 × 10^6^ lung cells (Fig. 2B), resulting in the hypothesis that residual BCG from the immunisation was also being quantified by the BACTEC MGIT system after the MGIA co-culture, falsely inflating CFU estimates and potentially masking vaccine responses. In our hands, BCG Pasteur Aeras grows faster than MTB Erdman when plated on 7H11 agar plates (data not shown). The BACTEC MGIT system is unable to differentiate between mycobacterial species and CFU input is estimated in the MGIA using a mycobacteria-specific standard curve. Therefore, we hypothesised that in the MTB Erdman MGIA, fast-growing residual BCG from the immunisation would be quantified as MTB Erdman, potentially causing an even larger overestimation of bacterial count than in the BCG MGIA.

Intra-assay variability was considerably higher in the control and BCG s.c. groups when using 100 CFU MTB Erdman compared with BCG in the MGIA (Supplementary Table S1). Based on these observations, two adaptions were made to the MTB Erdman MGIA: i) mycobacterial input was increased to 200 CFU; and ii) TCH was added to MGITs/plates to ensure the enumeration of MTB Erdman only. *Mycobacterium bovis*, of which BCG is an attenuated form, is sensitive to low concentrations of TCH (1–5 μg/mL) on solid growth media^35^. Since MTB and other mycobacteria are not sensitive to TCH at these concentrations^35^, the compound is routinely added to agar plates in MTB challenge studies which include an i.n. BCG study group^29,30^. We report that a concentration of 2 and 0.08 μg/mL TCH added to agar plates and MGITs, respectively, can be used to inhibit growth of BCG Pasteur Aeras but not MTB Erdman (Fig. 2C,D). It is important to note that TCH sensitivity of members of the MTBC compared with MTB laboratory strains is variable^28,36^ and we recommend that sensitivity be determined on a case-by-case basis when using clinical isolates.

Using the adapted lung MGIA protocol, an MGIA was performed using 1 × 10^6^ lung cells and 200 CFU MTB Erdman (Fig. 3). The assay was performed in the presence or absence of TCH. Significant growth inhibition of MTB Erdman was noted in all vaccinated groups compared with the unvaccinated group and intra-assay variability (CV) was reduced using 200 CFU MTB Erdman compared to 100 CFU (Supplementary Table S1). When TCH was added to MGITs, a further reduction in MTB Erdman CFU was observed in the BCG i.n group. Differences in MTB Erdman growth inhibition following vaccination were consistent with *in vivo* protection reported in the literature^24,30^, as well as with growth inhibition observed in the BCG MGIA performed in this study. Using a low-dose MTB aerosol challenge model, Copland *et al*. report enhanced control of MTB in murine lung boosted with spore-FP1 following BCG prime compared with BCG alone (0.70 vs 1.44 log_10_ CFU reduction vs PBS control)^24^. A number of mouse studies have demonstrated superior protection in the lung in challenge studies following BCG i.n. compared with BCG s.c. at comparable time points following vaccination^30,37,38^. While the MGIA aims to model vaccine protection observed in *in vivo* challenge studies, we do not expect log_10_ CFU values from the BACTEC MGIT system to match those obtained from challenge studies. The number of host cells and CFU associated with *in vivo* studies are different to the MGIA and we predict that the assay is not able to model the full complexity of an *in vivo* system. It is however encouraging to observe that results are consistent at the group level with existing literature. We suggest that future studies using the lung MGIA perform challenge studies in parallel to confirm that growth inhibition following vaccination translates to protection in the murine model.

Kolibab *et al*. previously demonstrated that results obtained by BACTEC MGIT and colony counting for the splenocyte MGIA were comparable. The BACTEC MGIT system is generally favoured over colony counting since CFU determination is slower and more labour intensive than the BACTEC method, and the absence of standardised reagents and element of subjectivity in manual counting may result in increased inter-assay variation^15^. Furthermore, the BACTEC MGIT system is unaffected by clumping and provides an accurate computer-generated readout based on validated technology^21^. However, reagents and equipment for the BACTEC MGIT system are costly. Therefore, to make the lung MGIA more accessible, particularly in resource-limited settings, MTB Erdman enumeration was also performed by culture on solid media. Under the optimised conditions described, comparable results were observed in all groups (Supplementary Fig. S2). However, consistent with previous reports, a reduction in the sensitivity of the MGIA when plating on solid media has been found between independent assay runs of the optimised lung MGIA presented here (Supplementary Fig. S4). Where differences in growth inhibition in the lung between vaccinated and non-vaccinated animals are small, for example when administering BCG s.c., a consistent reduction in MTB Erdman growth of <0.50 log_10_ CFU is noted using the BACTEC MGIT system; however, this difference is inconsistently found on plates in experiments performed in tandem with the BACTEC system. This observation suggests that the BACTEC MGIT system should be used when differences in growth inhibition are expected to be more conservative in the lung, for example when using systemic vaccine regimens, to ensure that smaller differences in growth are detected by the assay.

## Conclusions

We present an optimised preclinical MGIA protocol to assess the ability of host lung cells to inhibit mycobacterial growth in this organ following vaccination. We demonstrate that factors including host cell density, *ex vivo* co-culture CFU input, residual live BCG from vaccination and CFU enumeration method all significantly affect the reliability of the MGIA. Furthermore, we present optimised solutions to these challenges and show for the first time the utility of the MGIA to assess TB vaccine efficacy in the lung by virtue of growth inhibition of both BCG and MTB Erdman *ex vivo*.

In the future, the lung MGIA may be applied to refinement of vaccine dose or regimen prior to clinical studies and provide early insight into variations in protection against different lineages of the MTBC. Further optimisation of the assay is required for use with multiple MTBC lineages and *in vivo* challenge studies will need to be performed in parallel with the assay to confirm that growth inhibition following vaccination translates to protection. At present, it is encouraging to see that growth inhibition observed using various vaccination strategies is consistent with existing literature at the group level, indicating that the MGIA may represent a complementary tool to MTB challenge studies and immunogenicity measures for preclinical evaluation of TB vaccine strategies. The assay also represents a viable method for the reduction and refinement of animal studies in the TB field.

## Materials and Methods

### Ethics statement

Animal experimentation was performed under licence PPL 70/8043 (superseded by P6CA9EB8D) issued by the UK Home Office according to the Animals (Scientific Procedures) Act 1986 and approved by the London School of Hygiene & Tropical Medicine (LSHTM) Animal Welfare and Ethics Review Body. This manuscript was prepared in accordance with the ARRIVE guidelines^39^.

### Mycobacteria

BCG Pasteur Aeras was obtained from Aeras (Rockville MD, USA) as 500 μL frozen aliquots. MTB Erdman was obtained from BEI Resources (Manassas, VA, USA) as a 1 mL frozen glycerol stock. MTB Erdman stocks were grown to log phase at 150 rpm and 37 °C in Middlebrook 7H9 Broth (Yorlab, York, UK) supplemented with 10% OADC (Yorlab), 0.05% Tween-80 (Sigma, Gillingham, UK) and 0.2% glycerol (Sigma). All work using MTB Erdman was performed in an aerosol containment level-3 laboratory at LSHTM in accordance with guidance from the UK Advisory Committee on Dangerous Pathogens (ACDP). All mycobacterial stocks were stored at −80 °C.

### Animals

For immunisation experiments, female C57BL/6 mice (5–7 weeks old; weight 16–20 g) were obtained from Charles River UK and rested for at least five days before procedure start. Animals were housed in groups of 5–6 with access to food and water *ad libitum*. Animals were randomly assigned to cages on arrival by staff in the Biological Services Facility at LSHTM. Each cage was randomly assigned a group number on commencement of the study.

### Immunisation

BCG Pasteur Aeras was thawed at room temperature and diluted in physiological saline solution for irrigation (Baxter Healthcare, Newbury, UK) to a final concentration of 10^7^ CFU/mL. S.c. BCG Pasteur Aeras was administered as 100 µL (1 × 10^6^ CFU) into the right or left leg flap using a sterile 25 G needle. For i.n. immunisations, mice were anaesthetised by intraperitoneal injection of ketamine (50 mg/kg; Ketalar, Pfizer Itd, Walton Oaks, UK) and xylazine (10 mg/kg; Rompun, Bayer plc, Newbury, UK) in physiological saline solution for irrigation, followed by delivery of 50 μL (0.5 × 10^6^ CFU) BCG Pasteur Aeras equally administered between the two nostrils. Animals in the BCG s.c. + spore-FP1 boost arm were administered BCG Pasteur Aeras s.c. (as described) at week 0, followed by i.n. boosting at week 3 with spore-FP1. FP1 is a fusion protein constructed of MTB proteins, ACR, Ag85B and HBHA. Cloning and production of FP1 were previously described by Copland *et al*. For formulation of spore-FP1, FP1 (Lionex GmbH, Braunschweig, Germany) was incubated with *Bacillus subtilis* spores (Sporegen, Egham, UK) for one hour at room temperature and administered shortly following formulation. Animals were anaesthetised in the manner described for i.n. BCG, followed by delivery of 1 × 10^9^ *B. subtilis* spores coated with 10 µg FP1 (delivery volume: 40 µL/animal) equally between the two nostrils. Control animals received no treatment. BCG CFU input was confirmed by plating the diluted inoculum on Middlebrook 7H11 agar (Yorlab) plates containing 10% OADC supplement and 0.5% glycerol. All animals were rested for six weeks following vaccination and then sacrificed by exposure to carbon dioxide gas in a rising concentration.

### Lung and spleen cell isolation

Lungs were removed aseptically, washed in R0L (RPMI-1640 (Sigma) + 2 mM L-Glutamine [Fisher Scientific, Loughborough, UK] + 50 U/mL penicillin [Sigma]) and cut into small pieces with sterile scissors. Lungs were incubated for 40 minutes at 37 °C (5% CO_2_) with deoxyribonuclease I from bovine pancreas (type IV, Sigma; final concentration: 12 μg/mL) and Liberase TL (Sigma; final concentration: 625 μg/mL). The enzyme reaction was terminated by adding R10L (R0L + 10% heat-inactivated FBS [Sigma]) and homogenised lungs were passed through a 100-μm cell strainer (VWR International, Lutterworth, UK) to obtain a single cell suspension. Red blood cell lysis was performed using ammonium–chloride–potassium (ACK) lysis buffer. Spleens were removed aseptically and homogenised by passing through a 100-μm cell strainer. Red blood cell lysis was performed using RBC lysis buffer (Sigma). Processing of spleens was performed in R10S (antibiotic-free R10L). For all assays, samples from six mice were used to generate one pool per group.

### *Ex vivo* mycobacterial growth inhibition assay

Following cell isolation, murine cell number was counted, adjusted in R10 and 300 μL/well added to 48-well plates (Fisher Scientific. Loughborough, UK). A mastermix of the required bacterial input was made by diluting bacteria to the desired concentration in R10 and 300 μl added to each well containing cells. Plates were incubated at 37 °C (5% CO_2_) for 96 hours. At 96 hours, supernatants were removed from the plates and 500 μL sterile water was added to each well and incubated for five minutes. Murine cells were disrupted/lysed by pipetting to release intracellular mycobacteria and then the total volume (500 μL) was: i) added to a PANTA-supplemented MGIT (BD, Oxford, UK); or ii) plated on 7H11 agar plates supplemented with OADC and glycerol. ‘Direct-to-MGITs’ containing bacteria only were set up to confirm bacterial input, as well as for murine cells from immunised groups to detect residual BCG. All MGITs were placed in the BACTEC MGIT 960 or 320 systems (BD) until registered positive.

Standard curves were produced for BCG Pasteur Aeras and MTB Erdman (Supplementary Fig. S5) as described previously^27^ in order to convert TTP to bacterial count (CFU).

### *Mycobacterium* spp. 2-thiophenecarboxylic acid hydrazide susceptibility

To confirm susceptibility of BCG Pasteur Aeras and MTB Erdman to TCH in agar plates, 100 CFU BCG Pasteur Aeras and/or 100 CFU MTB Erdman were plated on 7H11 agar plates (supplemented with OADC and glycerol) in the absence or presence of 2 μg/mL TCH (Sigma). Five-fold serial dilutions of a 2 μg/mL TCH stock (0.4, 0.08 and 0.016 μg/mL) were performed and added to supplemented MGITs. Susceptibility of BCG Pasteur Aeras and MTB Erdman to TCH in MGITs was determined by adding 100 CFU BCG Pasteur Aeras and/or 100 CFU MTB Erdman to the MGITs. Tubes were placed in the BACTEC MGIT system until registered positive. CFU input was confirmed in the absence of TCH.

### Statistical analysis

All statistical analyses were carried out using GraphPad Prism Software version 7 (GraphPad, La Jolla, CA, United States). Data sets were found to follow Gaussian (normal) distribution using the D’Agostino-Pearson omnibus test. One-way analysis of variance (ANOVA) followed by Tukey’s multiple comparison test was used to determine whether there were any statistically significant differences between groups. A p value (adjusted) of <0.05 was considered statistically significant.

## Supplementary information

Supplementary information.

## Data Availability

Data will be shared on request.
