# Supplementary material for: Adaption of the ex vivo mycobacterial growth inhibition assay for use with murine lung cells
Source: Sci Rep. 2020 Feb 24;10:3311. doi: 10.1038/s41598-020-60223-y (PMC7039920; doi:10.1038/s41598-020-60223-y)
Supplement: Supplementary file 1 — Supplementary information. [file 41598_2020_60223_MOESM1_ESM.pdf]

Supplementary information

Title: Adaption of the *ex vivo* mycobacterial growth inhibition assay for use with murine lung cells

Authors: Hannah Painter, Satria A Prabowo, Felipe Cia, Lisa Stockdale, Rachel Tanner, Samuel Willcocks, Rajko Reljic, Helen A Fletcher and Andrea Zelmer

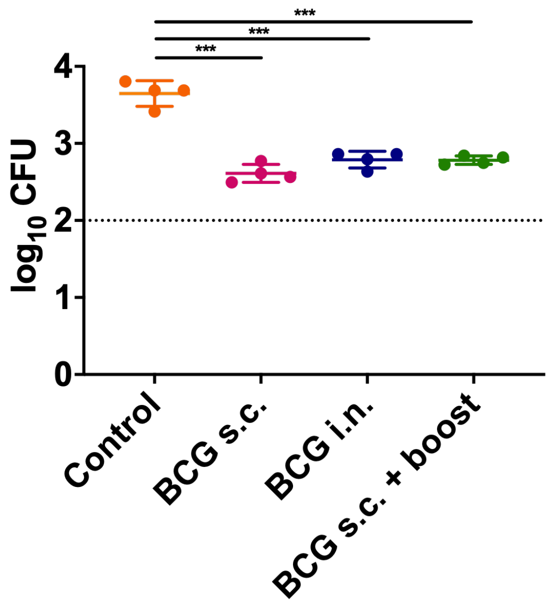

Figure S1. ***Ex vivo*** **MGIA comparing BCG growth inhibition conferred by vaccination in murine splenocytes.** C57BL/6 mice (n=6/group) received s.c. or i.n. BCG Pasteur Aeras at week 0, s.c. BCG Pasteur Aeras at week 0 and i.n. boosting with the candidate vaccine spore-FP1 at week 3, or received no treatment (control). At six weeks, 3×10^6 spleen cells were co-cultured with 100 CFU BCG Pasteur Aeras in 48-well plates. At 96 hours, samples were transferred to the BACTEC system until TTP values were generated. TTP values were converted to $\log_{10}$ CFU based on a standard curve. Data points (n=4/group) represent samples generated from pooled cells isolated from six mice. Dotted line indicates mycobacterial input at day 0. Statistical significance was tested by one-way ANOVA followed by Tukey's multiple comparison test. Error bars represent mean ± standard deviation. *** $p<0.001$ (adjusted). ANOVA, analysis of variance; BCG, Bacille Calmette-Guérin; CFU, colony-forming units; i.n., intranasal; MGIA, mycobacterial growth inhibition assay; s.c., subcutaneous; TTP, time to positivity.

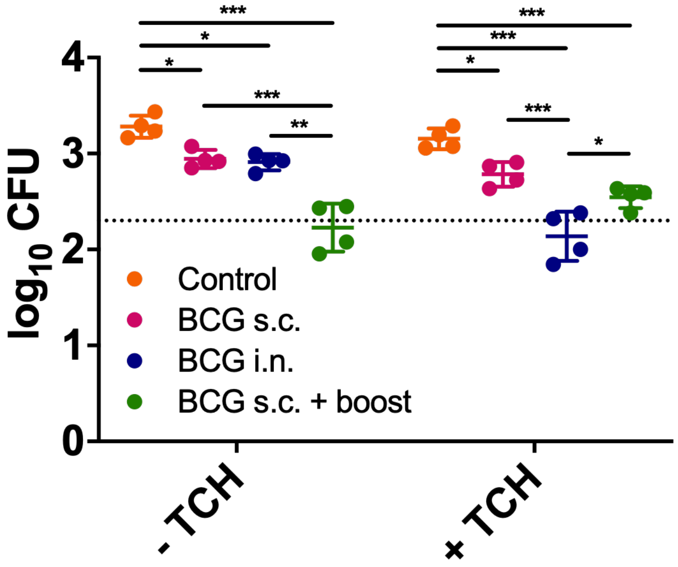

Figure S2. Quantification of MTB Erdman growth inhibition by plating on solid media in the optimised *ex vivo* MGIA. C57BL/6 mice (n=6/group) received s.c. or i.n. BCG Pasteur Aeras at week 0, s.c. BCG Pasteur Aeras at week 0 and i.n. boosting with the candidate vaccine spore-FP1 at week 3, or received no treatment (control). At six weeks, $1x10^6$ lung cells were co-cultured with 200 CFU MTB Erdman in 48-well plates. At 96 hours, samples were plated on 7H11 agar plates and incubated at 37°C for approximately three weeks. Mycobacterial quantification was performed in the presence and absence of TCH. Data points (n=4/group) represent samples generated from pooled cells isolated from six mice. Dotted line indicates mycobacterial input at day 0. Statistical significance was tested by one-way ANOVA followed by Tukey's multiple comparison test. Error bars represent mean ± standard deviation. * p<0.05 (adjusted); ** p<0.01 (adjusted); *** p<0.001 (adjusted). ANOVA, analysis of variance; BCG, Bacille Calmette-Guérin; CFU, colony-forming units; i.n., intranasal; MGIA, mycobacterial growth inhibition assay; s.c., subcutaneous; TCH, 2-thiophenecarboxylic acid hydrazide.

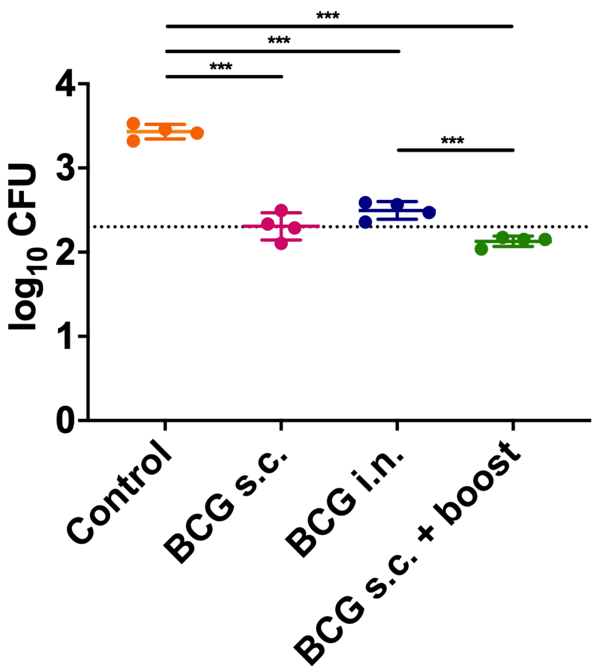

Figure S3. ***Ex vivo*** **MGIA comparing MTB Erdman growth inhibition conferred by vaccination in murine splenocytes.** C57BL/6 mice (n=6/group) received s.c. or i.n. BCG Pasteur Aeras at week 0, s.c. BCG Pasteur Aeras at week 0 and i.n. boosting with the candidate vaccine spore-FP1 at week 3, or received no treatment (control). At six weeks, $3x10^6$ spleen cells were co-cultured with 200 CFU MTB Erdman in 48-well plates. At 96 hours, samples were transferred to the BACTEC system until TTP values were generated. TTP values were converted to log_{10} CFU based on a standard curve. Data points (n=4/group) represent samples generated from pooled cells isolated from six mice. Dotted line indicates mycobacterial input at day 0. Statistical significance was tested by one-way ANOVA followed by Tukey's multiple comparison test. Error bars represent mean ± standard deviation. *** p<0.001 (adjusted). ANOVA, analysis of variance; BCG, Bacille Calmette-Guérin; CFU, colony-forming units; i.n., intranasal; MGIA, mycobacterial growth inhibition assay; s.c., subcutaneous; TTP, time to positivity

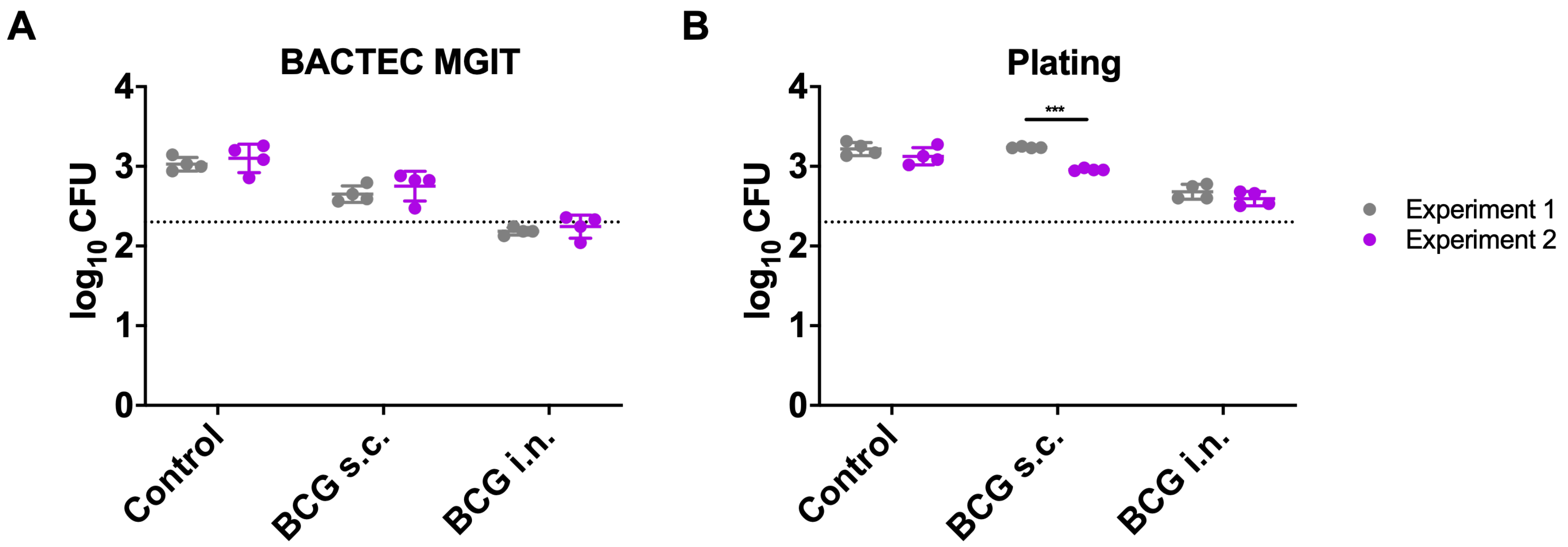

Figure S4. Comparison of inter-assay consistency of MTB Erdman growth inhibition between BACTEC MGIT and plating. C57BL/6 mice (n=6/group) received s.c. or i.n. BCG Pasteur Aeras at week 0 or received no treatment (control). At six weeks, $1x10^6$ lung cells were co-cultured with 200 CFU MTB Erdman in 48-well plates. At 96 hours, samples were (A) transferred to the BACTEC system until TTP values were generated or (B) plated on 7H11 agar plates and incubated at 37°C for approximately three weeks. TTP values were converted to log_{10} CFU based on a standard curve. Mycobacterial quantification was performed in the presence of TCH. Two independent assay repeats of the optimised lung MGIA (quantification by BACTEC MGIT system and plating) were performed from two separate mouse experiments. Data points (n=4/group) represent samples generated from pooled cells isolated from six mice. Dotted line indicates mycobacterial input at day 0. Statistical significance between the two experiment runs at the group level was tested by unpaired t-test. Error bars represent mean ± standard deviation. *** p<0.001 (adjusted). ANOVA, analysis of variance; BCG, Bacille Calmette-Guérin; CFU, colony-forming units; i.n., intranasal; MGIA, mycobacterial growth inhibition assay; MGIT, mycobacterial growth indicator tube; s.c., subcutaneous; TCH, 2-thiophenecarboxylic acid hydrazide.

A

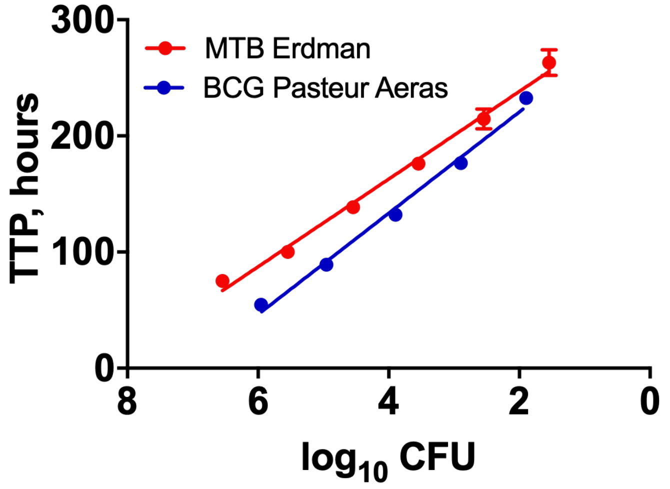

B

Mycobacterium spp.	Equation	R^2
MTB Erdman	Y = –37.74 * X + 313.8	0.9926
BCG Pasteur Aeras	Y = –43.59 * X + 307.8	0.9918

Figure S5. BACTEC MGIT standard curves. Serial ten-fold dilutions of MTB Erdman or BCG Pasteur Aeras were added to supplemented MGITs in duplicate to generate TTP values. Inoculum size was determined by plating the bacterial dilutions on 7H11 agar plates to calculate total CFU. (A) Total CFU was converted to $\log_{10}$ CFU and plotted against TTP. Error bars represent mean ± standard deviation . (B) Subsequent linear regression analysis was performed by fitting a semi-log line. R^2 value indicates goodness of fit. BCG, Bacille Calmette-Guérin; CFU, colony-forming units; MGIT, mycobacterial growth indicator tube; TTP, time to positivity.

	CV, %			
**MGIA**	**Control**	**BCG s.c.**	**BCG i.n.**	**BCG s.c. + boost**
**MTB Erdman BACTEC MGIT (100 CFU; –TCH)**	7.17	3.52	0.95	-
**MTB Erdman BACTEC MGIT (200 CFU; –TCH)**	2.48	1.96	0.89	0.83
**MTB Erdman BACTEC MGIT (200 CFU; +TCH)**	2.64	2.14	0.64	0.84

Table S1. MGIA intra-assay variability. C57BL/6 mice (n=6/group) received s.c. or i.n. BCG Pasteur Aeras at week 0, s.c. BCG Pasteur Aeras at week 0 and i.n boosting with the candidate vaccine spore-FP1 at week 3, or received no treatment (control). At six weeks, $1x10^6$ lung cells were co-cultured with MTB Erdman (CFU specified in rows) in 48-well plates. Four technical replicates per group were generated from pooled cells isolated from six mice. At 96 hours, samples were transferred to the BACTEC system until TTP values were generated. CV values were calculated for all groups under various MGIA conditions using raw TTP values (CV = (standard deviation/mean) x 100). BCG, Bacille Calmette-Guérin; CFU, colony-forming units; CV, coefficient of variation; i.n., intranasal; MGIA, mycobacterial growth inhibition assay; MGIT, mycobacterial growth indicator tube; s.c., subcutaneous; TCH, 2-thiophenecarboxylic acid hydrazide; TTP, time to positivity.
